# Association Between a Comprehensive Movement Assessment and Metabolically Healthy Overweight Obese Adults

**DOI:** 10.1038/s41598-020-58089-1

**Published:** 2020-01-24

**Authors:** Luke MacLeod, Danielle R. Bouchard, Jeffrey J. Hébert, Jonathan G. Boudreau, Martin Sénéchal

**Affiliations:** 1 Cardiometabolic Exercise & Lifestyle Laboratory, University of New Brunswick, 90 Mackay Drive, Fredericton, New Brunswick E3B 5A3 Canada; 2 Faculty of Kinesiology, University of New Brunswick, 90 Mackay Drive, Fredericton, New Brunswick E3B 5A3 Canada; 3 School of Psychology and Exercise Science, Murdoch University, 90 South Street, Murdoch, Western Australia Australia; 4 New Brunswick Institute for Research, Data and Training, University of New Brunswick, 38 Dineen Drive, 304F Keirstead Hall, Fredericton, New Brunswick E3B 5A3 Canada

**Keywords:** Epidemiology, Risk factors

## Abstract

Physical activity (PA) and sedentary behavior are associated with metabolic health in overweight and obese individuals. However, the role of comprehensive health-related movement guidelines on PA, recreational screen time, and sleep among Metabolically Healthy Overweight-Obese (MHO) individuals is unknown. We investigated differences in comprehensive movement assessment scores between adults classified as MHO or Non-MHO. The sample included 513 adults (46.2% male), aged 19 to 85 years, body mass index (BMI) ≥ 25, from cycle 2005–2006 of the National Health and Nutrition Examination Survey. Comprehensive movement assessment outcomes were defined as meeting modified 24-Hour Movement Guidelines criteria, with thresholds adapted for adults. 13.8% of participants were MHO (normal serum glucose, triglycerides, HDL-cholesterol, and systolic and diastolic blood pressure). Only 1.4% of MHO participants met all guidelines. MHO and Non-MHO participants had similar comprehensive movement assessment scores (MHO: 18.3% vs. Non-MHO: 10.9%; p = 0.072). MHO individuals had less continuous recreational screen time than Non-MHO individuals (1.8 ± 1.4 hrs/day vs. 2.5 ± 1.6 hrs/day; p < 0.001). Meeting the recreational screen time recommendation was the only variable associated with the MHO phenotype (OR:4.84 95%CI: 2.33–10.07). This association remained after adjusting for age, sex, ethnicity, education, and BMI (OR: 3.53 95%CI: 1.72–7.24). Our results suggest the importance of limiting recreational screen time in adults to optimize cardiometabolic risk profile in individuals living with overweight or obesity. Using movement guidelines with a screen time component to assess the risk associated with health outcomes in adults appears to provide a better assessment.

## Introduction

The increasing prevalence of obesity has prompted further research on the risks related to excessive adiposity. Obesity is associated with adverse cardiometabolic risk factors, including dyslipidemia, insulin resistance, hypertension, and metabolic syndrome^1,2^. However, some data suggests that a subgroup of overweight and obese individuals do not display typical cardiometabolic risk factors and are at lower risk of cardiovascular diseases and Type 2 diabetes^3^. These individuals are termed Metabolically healthy overweight-obese (MHO).

The prevalence of MHO varies between 7–74% in youth and adult populations, depending on the definition used^4–6^. MHO Individuals usually have a favorable metabolic profile, characterized by high insulin sensitivity, greater peripheral body fat, and low systemic inflammation, as well as normal blood pressure, lipid profile, and hepatic triglycerides content^1,7–9^. Physical activity (PA) has been associated with the MHO phenotype; however, a study has suggested that sedentary behavior, defined as any activity with an intensity of ≤1.5 Metabolic Equivalent Tasks, is an independent risk factor for the development of cardiometabolic risk factors^10,11^. In fact, substituting sedentary behavior with light PA is associated with decreased waist circumference, systolic and diastolic blood pressure, and increased high-density lipoprotein cholesterol (HDL-cholesterol)^10^. Replacing sedentary time with moderate-to-vigorous intensity physical activity (MVPA) is associated with reductions in clustered cardiometabolic risk factors^10^. Furthermore, a recent meta-analysis identified an association between sedentary time and all-cause mortality, independent of PA^11^. These results have been confirmed by other studies, suggesting that high amounts of sedentary time may counteract the benefits of MVPA^10–13^. Together, these findings point to a need for a more comprehensive evaluation of PA and its relationship with multiple movements and cardiometabolic risk factors.

The Canadian 24-Hour Movement Guidelines for children are promoted as a comprehensive movement assessment to tackle the increased prevalence of obesity, physical inactivity, sedentary time, and cardiometabolic risk factors among youth^14^. These guidelines are comprised of daily recommendations for sleep duration (9–11 hours of uninterrupted sleep for children ages 5–13 years, and 8–10 hours for ages 14–17 years), sitting time (less than 2 hours of recreational screen time), and PA intensities (60 minutes of daily MVPA, and several hours of LPA each day)^15–18^. There is a need to assess PA and movement in a more comprehensive way in all age groups. To date, a similar set of guidelines for adults and evidence of its association with cardiometabolic risk factors has not yet been established. Moreover, the associations between the guideline components and the MHO phenotype are unknown.

Therefore, we analyzed National Health and Nutrition Examination Survey (NHANES) data to examine for differences in comprehensive movement assessment scores between adults classified as MHO or Non-MHO. We hypothesized that individuals reaching the comprehensive movement assessment and its components would have a greater likelihood of being MHO.

## Methods

### Study population

**Figure 1:**
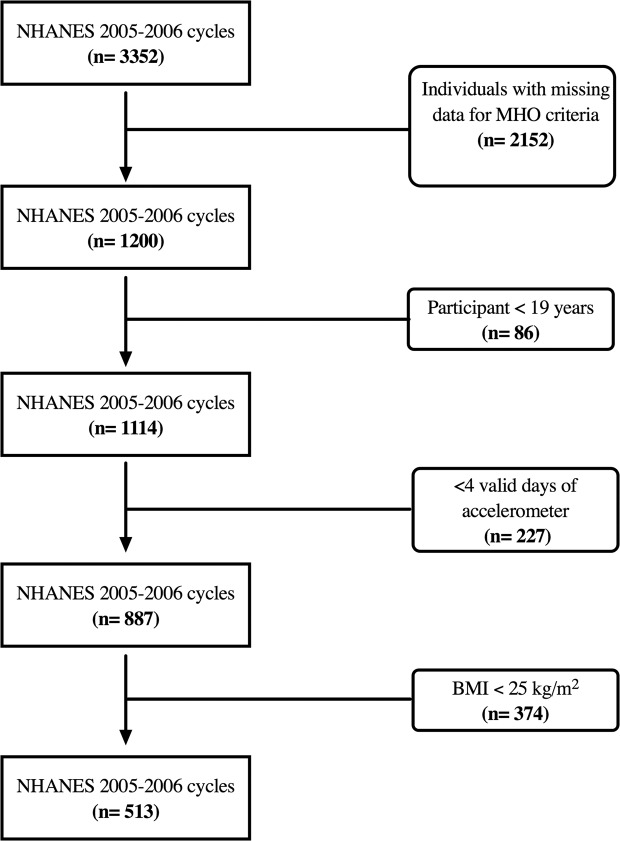
Final Sample Flow Chart.

The study sample consisted of 513 men and women who participated in the 2005–2006 cycles of the *National Health and Nutrition Examination Survey* (NHANES) and were between the ages of 19 and 85 years old. NHANES was approved by the National Center for Health Statistics institutional review board and written consent was obtained from participants. The original sample included 3352 adults before exclusions. Blood glucose, blood pressure, recreational screen time, and sleep data were missing for 1044 participants, while 1108 participants were missing HDL-cholesterol and triglyceride data. Of the remaining 1200 men and women, 86 did not meet the age requirement (≥19 years), and another 227 were missing accelerometer information. After applying accelerometer requirements to the sample (≥4 valid days) and removing individuals who had a body mass index (BMI) of ≤25, the sample size used for our analysis included 513 men and women (Fig. 1).

In NHANES, participants were identified with stratified multistage probability sampling. Detailed survey operation manuals and consent forms are available on the NHANES website^19^. Briefly, the NHANES survey consisted of a home interview and a thorough health examination. During the interview, participants were asked about their health status, disease history, and lifestyle behaviors. Health examinations were performed in a mobile exam center. The National Center for Health Statistics reviewed and approved the protocol (Protocol #2005–06). All participants provided written informed consent all experiments were performed in accordance with relevant guidelines and regulations.

## Primary Outcome

### Metabolically healthy Obese (MHO)

To categorize participants as MHO, the harmonized definition of metabolic syndrome from Alberti *et al*. (2009) was used^20^. Participants were considered MHO when meeting all of the following criteria: fasting glucose <5.6 mmol/L, triglycerides <1.7 mmol/L, HDL-cholesterol ≥1.0 mmol/L for males and ≥1.3 mmol/L for females, and systolic and diastolic blood pressure <130 mmHg and 85 mmHg, respectively, and therefore display the absence of cardiometabolic risk factors.

## Primary Exposure Variables

### Physical activity

Participants wore an accelerometer on their right hip that was fixed to an elastic belt and customized to each participant’s waist circumference. The accelerometer used was the ActiGraph (Model # Am-7164, manufactured by ActiGraph of Ft Walton Beach, Florida) and intensity readings were summed over each 1-minute epoch. Participants were instructed to wear the accelerometer during all waking hours for seven consecutive days and to remove it at night and during water-based activities (e.g., bathing, swimming). The data were considered valid if the device had been worn for a minimum of four days, with a minimum average wear time of 10 hours per day^21^. Non-wear time was identified by at least 60 consecutive minutes of counts between 0 and 100^21^. MVPA and light PA, were identified using age and sex specific cut-points^21^.

### Recreational screen time

Recreational screen time was self-reported during the household interview using the following question: “over the past 30 days, on average, how many hours per day did you sit and watch TV or videos?” Recreational screen time was estimated based on the answers to this question.

### Sleep

In order to align with the 24-Hour Movement Guidelines, self-reported number of hours of sleep per night was used in this study. Therefore, the following question was used: “How much sleep do you get (hours)?^22^”. Number of hours of sleep ranged between 1 to 11 hours, while values ≥12 hours were categorized as 12 hours or more^22^.

### Comprehensive movement assessment

The 24-Hour Movement Guidelines from the Canadian Society for Exercise Physiology were adapted to create a comprehensive movement assessment. The guidelines include recommendations for PA, sleep, and sitting time. The PA component aligns with the adult Canadian Physical Activity guidelines that recommend at least 150 minutes of MVPA per week occurring in 10-minute bouts^23^. The 24-Hour Movement Guidelines further stipulate that individuals should perform several hours of structured and unstructured light PA, but they do not give a specific recommendation for the number of hours. Therefore, time in light PA was quantified, and quartiles for the study sample were computed. The highest quartile was used as the criterion for reaching this component of the comprehensive movement assessment. The sleep recommendation for adults should be between 7 and 9 hours^24^. Therefore, participants sleeping between 7 to 9 hours per night were considered to be meeting the sleep guideline, while participants sleeping either <7 hours or >9 hours per night were considered to be not meeting this guideline component. As for recreational screen time, participants were found to meet this component of the comprehensive movement assessment if they had ≤2 hours of recreational screen time per day. Recreational screen time was measured using the number of hours spent watching television per day. Therefore, a single dichotomous variable was created based on MVPA, light PA, sleep, and recreational screen time. Thereafter, participants were categorized as meeting or not meeting the guidelines based on this comprehensive assessment.

### Anthropometric measures

Height, weight, waist circumference, and BMI measures were taken by following the protocol outlined by the Anthropometric Standardization Reference Manual^25^. All examination procedures were obtained by trained health technicians^26^.

### Covariates

Covariates included age, sex, ethnicity, and educational level. This information was collected through in-person household interviews. Categories were created for ethnicity, including Hispanic, non-Hispanic white, non-Hispanic black, and other. Education levels were categorized as less than high school (lower than 9 grade-12 grades), high school (graduate or equivalent), or more than high school (at least of a college degree).

### Statistical analysis

Exposure and outcome variables were described using mean ± standard deviation (SD) for continuous variables and frequency n (%) for categorical variables. Confounding variables that were not normally distributed were reported as median and interquartile range (IQR). Independent t-tests and chi-Square tests were performed to identify between-group differences among participants classified as MHO or Non-MHO. Multivariable logistic regression models were used to investigate the independent associations between the comprehensive movement assessment components, as continuous variables and as dichotomous variables indicating whether or not the guidelines were met, and MHO status. Each individual’s dichotomous variable was combined into a composite score to evaluate individuals that meet the comprehensive movement assessment guidelines and to investigate its association with MHO and Non-MHO. All models were adjusted for age, gender, ethnicity, education, and BMI. Data management and statistical analyses were performed using SPSS for Windows version 20 and SAS software version 9.4 for Windows (SAS Institute Inc., Cary, NC, USA, Copyrights^©^ 2012). An alpha level of 0.05 was used for all analyses. Statistics accounted for the sample weights and complex survey design (strata, probability sampling units).

## Results

Table 1 describes the characteristics of the study sample. Overall, 53.0% were non-Hispanic white and 46.2% were men, with mean ± SD age of 51.4 ± 18.4 years. The averages for BMI and waist circumference were 31.6 ± 5.6 kg/m^2^ and 106.0 ± 13.2 cm respectively, while average systolic and diastolic blood pressure were 128.4 ± 21.1 mmHg and 70.4 ± 12.8 mmHg respectively. Mean fasting blood glucose was 6.1 ± 2.0 mml/L, while triglyceride and HDL-cholesterol averages were 2.0 ± 1.0 mmol/L and 1.4 ± 0.3 mmol/L. The proportion of MHO and Non-MHO was 13.8% and 86.2%, respectively.

**Table 1:** Descriptive characteristics.

	MHO (N = 71)	Non-MHO (N = 442)	P-Value
***General characteristics***			
Men n (%)	33 (46.5)	204 (46.2)	0.959
Age (years)	42.7 ± 16.0	52.9 ± 17.5	0.000
BMI (kg/m^2^)	28.3 [26.3–31.3]	30.3 [28.0–34.6]	0.000
Waist circumference (cm)	100.1 ± 11.0	107.1 ± 13.3	0.000
***Ethnicity***			
Non-Hispanic white n (%)	29 (40.8)	243 (55.0)	0.026
Non-Hispanic black n (%)	20 (28.2)	69 (15.6)	
Hispanic n (%)	21 (29.6)	113 (25.6)	
Other n (%)	1 (1.4)	17 (3.8)	
***Education***			
Middle School	11 (15.5)	133 (30.1)	0.033
High School	41 (57.7)	199 (45.0)	
***Metabolic Variables***			
Systolic blood pressure (mmHg)	113.8 ± 9.5	130.8 ± 21.5	0.000
Diastolic blood pressure (mmHg)	68.3 ± 9.7	70.8 ± 13.3	0.063
Fasting glucose (mmol/L)	5.1 ± 0.3	6.2 ± 2.1	0.000
Triglyceride (mmol/L)	1.3 ± 0.2	2.1 ± 1.0	0.000
HDL-cholesterol (mmol/L)	1.5 ± 0.4	1.4 ± 0.3	0.002

Data are presented as mean ± SD or median [IQR] for continuous variables and N (%) for categorical variables,

MHO = Metabolically healthy overweight-obese, BMI = Body mass index, HDL = High density lipoprotein.

There were significant differences between MHO and Non-MHO individuals for BMI (29.4 ± 4.2 vs. 31.95 ± 5.8; p = 0.0001) and waist circumference (100.1 ± 11.0 vs. 107.1 ± 13.3; p = 0.0001). Compared to Non-MHO, ethnicity and education level were all significantly different (p > 0.05). As for metabolic profile, MHO individuals had lower systolic blood pressure (113.8 ± 9.5 vs. 130.8 ± 21.5 mmHg; p = 0.0001), blood glucose (5.1 ± 0.3 vs. 6.2 ± 2.1 mmol/L; p = 0.0001), and triglycerides (1.3 ± 0.2 vs. 2.1 ± 1.0 mmol/L; p = 0.0001), and had higher HDL-cholesterol (1.5 ± 0.4 vs. 1.4 ± 0.3 mmol/L; p = 0.002).

Table 2 describes each component of the comprehensive movement assessment and shows the proportion of individuals that met the criteria stratified by MHO status. Overall, MHO individuals had less recreational screen time compared to Non-MHO (1.8 ± 1.4 hrs/day vs. 2.5 ± 1.6 hrs/day; p = 0.0001), while no differences between groups were observed for MVPA, light PA, or sleep (p > 0.05). Compared to the Non-MHO group, the MHO group had the greatest proportion of individuals who met the guideline for recreational screen time (76.1% vs. 55.4%; p = 0.001). No differences were observed for the proportion of individuals meeting the guideline for MVPA, light PA, or sleep (p > 0.05).

**Table 2:** Comprehensive Movement Assessment Differences among MHO and Non-MHO participants.

	MHO (N = 71)	Non-MHO (N = 442)	P-Value
	**Continuous Variables**		
***Moderate to Vigorous Physical Activity***			
Total MVPA per week (min/week)	46.5 ± 81.1	33.6 ± 76.1	0.200
***Light Physical Activity***			
Total light PA (min/week)	1824.6 ± 435.8	1775.0 ± 496.0	0.427
***Sleep***			
Total sleep time (hours/night)	6.9 ± 1.4	6.8 ± 1.5	0.575
***Recreational Screen Time***			
Total recreational screen time (hours/day)	1.8 ± 1.4	2.5 ± 1.6	<0.001
	**Proportion of Individual Meeting Each Component**		
***Physical Activity***			
≥150 min/week of MVPA n (%)	6 (8.5)	33 (7.5)	0.771
***Light Physical Activity***			
High light PA (min/week) n (%) *	22 (31.0)	107 (24.2)	0.222
***Sleep***			
7–9 hours/night n (%)	41 (57.7)	261 (59.0)	0.836
***Recreational Screen Time***			
≤2 hours/day n (%)	54 (76.1)	245 (55.4)	0.001
	**Comprehensive Movement Assessment**		
**Number of Components**			
Met all 4 guidelines n (%)	1 (1.4)	0 (0.0)	—
Met ≥ 3 criteria of the guidelines n (%)	13 (18.30)	48 (10.9)	0.072
Met ≥ 2 of 4 guidelines n (%)	44 (62.0)	213 (48.2)	0.031
Met ≥ 1 criteria of the guidelines n (%)	65 (91.5)	385 (87.1)	0.289

Data are presented as unweighted mean ± SD for continuous variables and N (%) for categorical variable and analyses were performed using the survey weights, MHO = Metabolically healthy overweight-obese, MVPA = moderate-to-vigorous physical activity, PA = physical activity, *Highest Quartile for light Physical Activity.

Table 3 describes the independent association between each component of the comprehensive movement assessment and MHO status. Total light PA and total MVPA were not significantly associated with the MHO phenotype (OR: 1.00 95%CI: 0.98–1.02 and OR: 0.94 95%CI: 0.86–1.04, respectively) even after adjustment (OR: 1.00 95%CI: 0.98–1.02 and OR: 1.03 95%CI: 0.89–1.20, respectively). Total sleep time was not significantly associated with the MHO phenotype (OR:0.91 95%CI: 0.71–1.17 after adjustment). However, total recreational screen time was significantly associated with MHO phenotype (OR:1.57 95%CI: 1.29–1.90). This relationship persisted after adjusting for age, sex, ethnicity, education, and BMI (OR:1.48 95%CI: 1.19–1.84).

**Table 3:** Logistic Regressions of Components of the Comprehensive Movement Assessment for MHO and Non-MHO.

	Model #1	Model #2
	**Continuous Variables**	
***Physical Activity***		
Total MVPA per week (30 min/week)	0.94 (0.86–1.04)	1.03 (0.89–1.20)
***Light Physical Activity***		
Total Light PA (30 min/week)	1.00 (0.98–1.02)	1.00 (0.98–1.02)
***Sleep***		
Total sleep time (hours/night)	0.90 (0.75–1.08)	0.91 (0.71–1.17)
***Recreational Screen Time***		
Total recreational screen time (hours/day)	1.57 (1.29–1.90)	1.48 (1.19–1.84)

Data presented as OR and (95% CI); MVPA = moderate-to-vigorous physical activity; Model #1 is unadjusted; Model #2 is adjusted for age, sex, ethnicity, education, and BMI. All analyses were performed using the survey weights.

**Figure 2:**
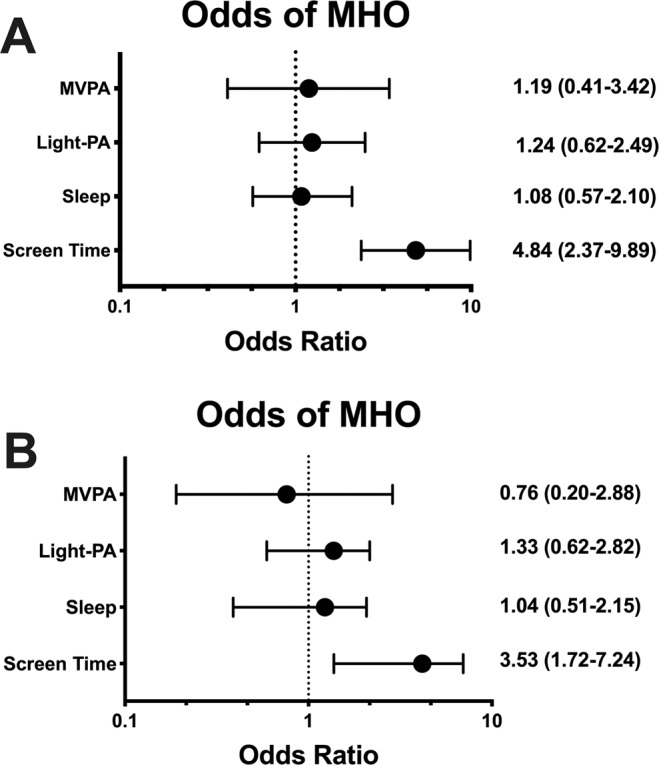
(**A,B**) Association between individual components of the comprehensive movement assessment, expressed as dichotomous variables. **A** = unadjusted; **B** = adjusted.

Figure 2A revealed that in an unadjusted model, meeting the threshold for MVPA (OR:1.19 95%CI: 0.41–3.42) or sleep time (OR:1.08 95%CI: 0.57–2.10) was not significantly associated with the MHO phenotype. Reduced recreational screen time was significantly associated with the likelihood of being MHO (OR: 4.84 95%CI: 2.37–9.89). This association remained significant after adjusting for confounders, including age, sex, and ethnicity (OR: 4.81 95%CI: 2.37–9.75). Further adjustment for education and BMI did not change this association between recreational screen time and the MHO phenotype (Fig. 2B; OR: 3.53 95%CI: 1.72–7.24).

**Figure 3:**
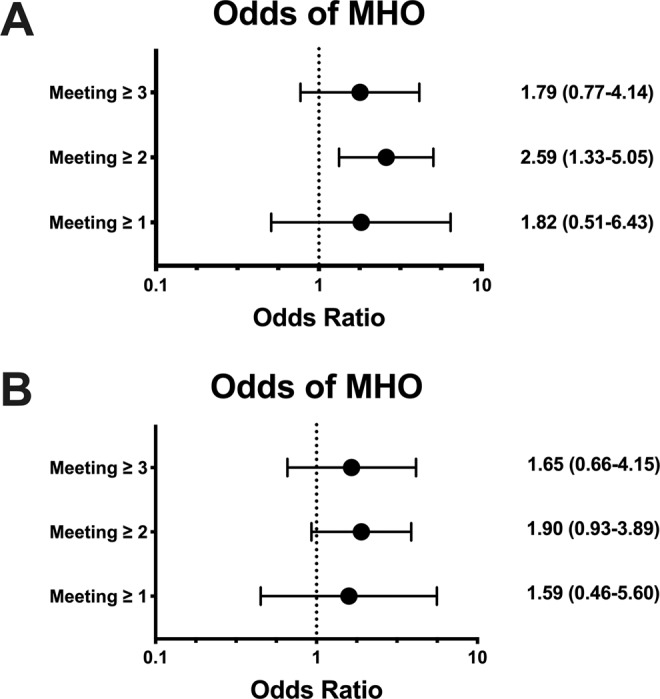
(**A**,**B**) Association between the number of comprehensive movement assessment criteria met and MHO phenotype. **A** = unadjusted; **B** = adjusted.

The proportion of MHO who reached the comprehensive movement assessment was 1.4%, while none of the Non-MHO individuals reached this guideline. The proportion of those who met at least 3 of the criteria was 18.3% for MHO, and 10.9% for Non-MHO (p > 0.072). Meeting one or more of the comprehensive movement assessment criteria was not associated with the MHO phenotype (Fig. 3A; OR: 1.82 95%CI: 0.51–6.43). However, meeting at least two criteria was significantly associated with the MHO phenotype (OR: 2.59 95%CI: 1.33–5.05). This association remain after adjustment for age, sex, ethnicity (OR: 2.45 95%CI: 1.23–4.90), but it was no longer significant after further adjustment for education and BMI (Fig. 3B; OR: 1.90 95%CI: 0.93–3.89).

## Discussion

The purpose of this study was to determine if there were independent associations between a comprehensive movement assessment and its components and the MHO phenotype among overweight and obese adults. It was hypothesized that those who were reaching the comprehensive movement assessment and its components would have a greater likelihood of being MHO compared to Non-MHO adults. Although previous studies have looked at the associations between meeting comprehensive movement guidelines and the likelihood of obesity^27,28^, this is the first study, to the best of our knowledge, to apply a comprehensive movement assessment to a sample of overweight and obese adults using the MHO phenotype as an outcome. The current study has many relevant results from a cardiometabolic perspective. First, about only 1% of our sample was meeting all of the components of the comprehensive movement assessment, which included 150 min of MVPA in 10-minute bouts, several hours of structured and non-structure light PA, less than 2 hours per day of recreational screen time, and 7 to 9 hours of sleep per night. Second, recreational screen time was the only component of the comprehensive movement assessment associated with the MHO phenotype after adjusting for all of the potential confounders. These findings are relevant from a cardiometabolic perspective as they reinforce the importance of limiting recreational screen time, even in adults, in order to reduce the likelihood of exhibiting cardiometabolic risk factors.

The primary finding of this study was the association observed between recreational screen time and the MHO phenotype. In fact, meeting the recreational screen time recommendation was associated with about a 4-fold increased likelihood of being MHO compared to Non-MHO, even after adjustment for confounders. However, meeting sleep, light PA, and MVPA recommendations were not related to likelihood of being MHO. These findings contrast those of a previous study on MHO and Non-MHO individuals, which suggested that PA, but not screen time, was significantly different between MHO and Non-MHO adults^29^. Potential explanations for these conflicting results could be the use of different definitions for MHO. For example, the study by Camhi *et al*. 2013 defined MHO as exhibiting one or fewer cardiometabolic risk factors, while our study stipulated that MHO individuals would not have any of these risk factors^29^. However, our results are supported by other findings showing that reduced sedentary behavior is an independent risk factor for developing cardiometabolic risk factors^10,11^. A systematic review of longitudinal studies on sedentary behavior found that sedentary time was consistently associated with greater risk of all-cause mortality and CVD-related mortality, regardless of PA levels and BMI^11^. In this study, meeting the recreational screen time guideline or having reduced recreational screen time, a proxy measure of sedentary behavior, was consistently associated with increased odds of being MHO, which suggests that minimizing sedentary behavior is associated with better cardiometabolic health compared to that of individuals not meeting the recommendations. This result is in accordance with other studies, which showed that screen time was associated with cardiometabolic risk factors and premature mortality^30^. In fact, spending ≥2.1 hours per day using screens was associated with an 80% increased odds of presenting cardiometabolic risk factors^31^. Another study determined that ≥2 hours of screen time per day doubled the likelihood of CVD events in men, compared to <2 hours of screen time^30^.This relationship was independent of physical activity. Interestingly, Knaeps *et al*. (2017) found that replacing 30 minutes of sedentary behavior with 30 minutes of light PA, MVPA, or sleep can lead to further cardiometabolic health benefits^10^.

Another finding of this study was that meeting at least two of the four components of the comprehensive movement assessment were associated with an increased likelihood of being MHO compared to Non-MHO individuals. One study found a dose-response relationship between the number of movement guidelines met and improvement in physical, mental, and social health outcomes, such as MVPA, screen time, prosocial behaviours, and life satisfaction, among children^32^. Similarly, Roman-Vinas *et al*. (2016) observed that children show a dose-response relationship between meeting movement guidelines and reduced risk of being obese^18^. Yet in our study, a dose-response relationship between guideline adherence and odds of being MHO among adults was not observed. These results could suggest that, for adults, meeting at least two components is associated with lower odds of presenting certain cardiometabolic risk factors, while meeting three or more is not associated with additional reduction in odds. This type of trend is seen in the relationship between PA and all-cause mortality, where there are greater differences in likelihood reduction between individuals doing low levels of activity and those doing moderate levels of activity than between moderately and highly active persons^33^. However, the association between meeting two or more criteria and MHO status lost significance when BMI was adjusted for, meaning that the relationship observed in the other two models could have been due to BMI differences among the sample.

While recreational screen time was independently associated with the MHO phenotype, neither light PA or MVPA were significantly associated with the MHO phenotype. This suggest that the amount of time spent in PA, at least on its own, is not necessarily associated with better cardiometabolic health. These results are surprising, considering the large body of evidence suggesting that PA is a strong predictor of cardiometabolic health^31,34–37^. For example, individuals who did not meet the MVPA guideline (150 minutes of MVPA in 10-minute bouts) were shown to have three times the odds of exhibiting cardiometabolic risk factors^38^.The lack of association between MHO status and MVPA could be due to the average amount of MVPA being quite low in both groups. As shown in Table 2, both MHO and Non-MHO individuals that were reaching close to the mean MVPA per week would have been doing well below recommended amount of 150 minutes of MVPA per week. Furthermore, those within one standard deviation of the group mean would also have failed to reach the guidelines. It is logical to believe that these levels of MVPA are insufficient to contribute to cardiometabolic health. Another factor that could have contributed to the absence of association was not including cardiorespiratory fitness in the MHO definition, which one study suggested doing, as cardiorespiratory fitness has been shown to attenuate cardiometabolic related risk among MHO individuals^39,40^. Contrary results from our group suggest no significant difference between MHO and Non-MHO adults for bouts of MVPA once confounders were adjusted for^41^. Furthermore, perhaps fitness is a better predictor of MHO likelihood and cardiometabolic risk than time spent in physical activity, as several studies have found that MHO individuals have higher fitness levels than their metabolically unhealthy counterparts^1,6,42^. Nevertheless, combining these findings with the association between MHO status and recreational screen time supports the idea of sedentary behavior being a risk factor for CVD, independent of PA^30^. With MHO status and other cardiometabolic risk factors being influenced by more variables than just PA, taking a more comprehensive approach to movement assessment is of great importance.

Although this study revealed important findings, several limitations need to be discussed. First, the cross-sectional design of the study does not allow for cause-and-effect relationships to be concluded. Second, the recreational screen time and sleep data were both self-reported; therefore, having more objective data may have influenced our results. Third, sedentary time was quantified using recreational screen time, despite accelerometers being available, which might not accurately reflect sedentary time; however, the rational for such this decision was to ensure that the defined 24-Hour Movement Guidelines were tested. Fourth, light PA was arbitrarily based on quartile, which may also have impacted our results. Fifth, medication was not accounted into the categorization of MHO, which could have impacted our results. Finally, the sample sized used for this study was quite small, but it allowed us to use a more comprehensive MHO definition. Although these limitations may have impacted our results, the study was strengthened by using a comprehensive definition of MHO, using an objective measure of MVPA, performing a weighted analysis, which accounted for the complex survey and design of the study, and adjusting for confounders. These aspects served to increase the external validity of the results.

In summary, recreational screen time was associated with the MHO phenotype in adults, while none of the other elements of the comprehensive movement assessment were associated with cardiometabolic health. The results of our study strengthen the importance of considering recreational screen time as another modifiable behavioral factor to protect against cardiometabolic health in adults. Future research should take a longitudinal and experimental approach and incorporate more objective methods of measuring sleep and recreational screen time.

## References

[1] Ortega FB (2013). The intriguing metabolically healthy but obese phenotype: cardiovascular prognosis and role of fitness. Eur. heart J..

[2] Sénéchal M, Bouchard DR, Dionne IJ, Brochu M (2012). Lifestyle habits and physical capacity in patients with moderate or severe metabolic syndrome. Metab. Syndr. Relat. Disord..

[3] Appleton SL (2013). Diabetes and cardiovascular disease outcomes in the metabolically healthy obese phenotype: a cohort study. Diabetes Care.

[4] Heinzle S, Ball GD, Kuk JL (2016). Variations in the prevalence and predictors of prevalent metabolically healthy obesity in adolescents. Pediatric Obes..

[5] van Vliet-Ostaptchouk JV (2014). The prevalence of metabolic syndrome and metabolically healthy obesity in Europe: a collaborative analysis of ten large cohort studies. BMC Endocr. Disord..

[6] Messier V (2010). Identifying metabolically healthy but obese individuals in sedentary postmenopausal women. Obes..

[7] Bluher M (2010). The distinction of metabolically ‘healthy’ from ‘unhealthy’ obese individuals. Curr. Opin. Lipidol..

[8] Brochu M (2001). What are the physical characteristics associated with a normal metabolic profile despite a high level of obesity in postmenopausal women?. J. Clin. Endocrinol. Metab..

[9] Sénéchal M (2013). Cardiorespiratory fitness and adiposity in metabolically healthy overweight and obese youth. Pediatrics.

[10] Knaeps S (2017). Substituting Sedentary Time With Light And Moderate-to-Vigorous Physical Activity is Associated With Better Cardio-Metabolic Health. J. Phys. Act. Health.

[11] Thorp AA, Owen N, Neuhaus M, Dunstan DW (2011). Sedentary behaviors and subsequent health outcomes in adults a systematic review of longitudinal studies, 1996–2011. Am. J. Prev. Med..

[12] Patel AV (2010). Leisure time spent sitting in relation to total mortality in a prospective cohort of US adults. Am. J. Epidemiol..

[13] Katzmarzyk PT, Church TS, Craig CL, Bouchard C (2009). Sitting time and mortality from all causes, cardiovascular disease, and cancer. Med. Sci. Sports Exerc..

[14] Tremblay MS (2016). Canadian 24-Hour Movement Guidelines for Children and Youth: An Integration of Physical Activity, Sedentary Behaviour, and Sleep. Appl. Physiol. Nutr. Metab..

[15] Chaput JP (2016). Systematic review of the relationships between sleep duration and health indicators in school-aged children and youth. Appl. Physiol. Nutr. Metab..

[16] Carson V (2016). Systematic review of sedentary behaviour and health indicators in school-aged children and youth: an update. Appl. Physiol. Nutr. Metab..

[17] Saunders TJ (2016). Combinations of physical activity, sedentary behaviour and sleep: relationships with health indicators in school-aged children and youth. Appl. Physiol. Nutr. Metab..

[18] Roman-Vinas B (2016). Proportion of children meeting recommendations for 24-hour movement guidelines and associations with adiposity in a 12-country study. Int. J. Behav. Nutr. Phys. Act..

[19] National Health and Nutrition Examination Survey (HANES), https://wwwn.cdc.gov/nchs/nhanes/ContinuousNhanes/overview.aspx?BeginYear=2005, Accessed October 24th 2019.

[20] Alberti KG (2009). Harmonizing the metabolic syndrome: a joint interim statement of the International Diabetes Federation Task Force on Epidemiology and Prevention; National Heart, Lung, and Blood Institute; American Heart Association; World Heart Federation; International Atherosclerosis Society; and International Association for the Study of Obesity. Circulation.

[21] Troiano RP (2008). Physical activity in the United States measured by accelerometer. Med. Sci. Sports Exerc..

[22] National Health and Nutrition Examination Survey (HANES): 2005–2006 Data Documentation, Codebook, and Frequencies, https://wwwn.cdc.gov/Nchs/Nhanes/2005-2006/SLQ_D.htm#SLD010H, Accessed October 24th 2019.

[23] Tremblay MS (2011). New Canadian physical activity guidelines. Appl. Physiol. Nutr. Metab..

[24] Chaput JP, Wong SL, Michaud I (2017). Duration and quality of sleep among Canadians aged 18 to 79. Health Rep..

[25] Lohman, T. G., Roche, A. F. *Anthropometric Standardization Reference Manual*. Human Kinetics, Champaingn III, 0–184, (Reynaldo Martorell, 1988).

[26] National Health and Nutrition Examination Survey (NHANES): *National Health and Nutrition Examination Survey: Data Documentation, Codebook, and Frequencies*, https://wwwn.cdc.gov/Nchs/Nhanes/2005-2006/BMX_D.htm, Accessed October 24th 2019.

[27] Katzmarzyk PT, Staiano AE (2017). Relationship Between Meeting 24-Hour Movement Guidelines and Cardiometabolic Risk Factors in Children. J. Phys. Act. Health.

[28] Lee EY (2017). Meeting new Canadian 24-Hour Movement Guidelines for the Early Years and associations with adiposity among toddlers living in Edmonton. Canada. BMC Public. Health.

[29] Camhi SM, Waring ME, Sisson SB, Hayman LL, Must A (2013). Physical activity and screen time in metabolically healthy obese phenotypes in adolescents and adults. J. Obes..

[30] Stamatakis E, Hamer M Fau - Dunstan DW, Dunstan DW (2011). Screen-based entertainment time, all-cause mortality, and cardiovascular events: population-based study with ongoing mortality and hospital events follow-up. J. Am. Coll. Cardiol..

[31] Rao DP, Orpana H, Krewski D (2016). Physical activity and non-movement behaviours: their independent and combined associations with metabolic syndrome. Int. J. Behav. Nutr. Phys. Act..

[32] Janssen I, Roberts KC, Thompson W (2017). Is adherence to the Canadian 24-Hour Movement Behaviour Guidelines for Children and Youth associated with improved indicators of physical, mental, and social health?. Appl. Physiol. Nutr. Metab..

[33] Office of Disease Prevention and Health Promotion, *Physical Activity Guidelines Advisory Committee Report, Part G. Section 1: All-Cause Mortality*, https://health.gov/paguidelines/report/G1_allcause.aspx#q2c, Accessed October 4th 2019.

[34] Velez-Toral M Fau - Godoy-Izquierdo D (2017). Improvements in Health-Related Quality of Life, Cardio-Metabolic Health, and Fitness in Postmenopausal Women After an Exercise Plus Health Promotion Intervention: A Randomized Controlled Trial. J. Phys. Act. Health.

[35] Swift DL, Earnest CP, Blair SN, Church TS (2012). The effect of different doses of aerobic exercise training on endothelial function in postmenopausal women with elevated blood pressure: results from the DREW study. Br. J. Sports Med..

[36] Swift DL (2012). The effect of exercise training modality on serum brain derived neurotrophic factor levels in individuals with type 2 diabetes. PLoS One.

[37] Swift DL (2012). Exercise training and habitual physical activity: a randomized controlled trial. Am. J. Prev. Med..

[38] Clarke J, Janssen I (2013). Is the frequency of weekly moderate-to-vigorous physical activity associated with the metabolic syndrome in Canadian adults?. Appl. Physiol. Nutr. Metab..

[39] Ortega FB, Cadenas-Sanchez C, Sui X, Blair SN, Lavie CJ (2015). Role of Fitness in the Metabolically Healthy but Obese Phenotype: A Review and Update. Prog. Cardiovasc. Dis..

[40] Jae SY (2017). Impact of Cardiorespiratory Fitness and Risk of Systemic Hypertension in Nonobese Versus Obese Men Who Are Metabolically Healthy or Unhealthy. Am. J. Cardiol..

[41] de Winter M, Rioux BV, Boudreau JG, Bouchard DR, Sénéchal M (2018). Physical Activity and Sedentary Patterns among Metabolically Healthy Individuals Living with Obesity. J. Diabetes Res..

[42] Gregorio-Arenas E (2016). The associations between physical fitness and cardiometabolic risk and body-size phenotypes in perimenopausal women. Maturitas.

